# Abortion Clients of a Public-sector Clinic and a Non-governmental Organization Clinic in Nepal

**DOI:** 10.3329/jhpn.v31i3.16830

**Published:** 2013-09

**Authors:** Shyam Thapa, Shailes Neupane

**Affiliations:** ^1^Nepal Public Health Foundation, Kathmandu, Nepal; ^2^Valley Research Group, Lalitpur, Nepal

**Keywords:** Abortion, Contraception, NGO clinic, Public clinic, Nepal

## Abstract

This paper investigates similarities and differences between abortion clients of a public-sector clinic and a non-governmental organization (NGO) clinic in Nepal. In 2010, a survey of 1,172 women was conducted in two highly-attended abortion clinics in Kathmandu—one public-sector clinic and another operated by an NGO. Data on the sociodemographic characteristics of clients, their fertility preferences, and use of contraceptives were analyzed. Similarities and differences between the two groups of clients were examined by either chi-square or *t*-test. The clients of the two clinics were similar with respect to age (27.3±5.7 years), education (26.5% had no education), and number of living children (1.88±1.08). They differed with regard to contraceptive practice, the circumstances resulting in unintended pregnancy, and future fertility preferences. Just over 50% clients of the public and 35% clients of the NGO clinic reported use of contraceptives surrounding the time of unintended pregnancy. The groups also differed in the contraceptive methods used and in reasons for not using any method. The NGO clinic contributed principally to expanding the availability of and access to abortion services.

## INTRODUCTION

Recent years have witnessed increased role of the private sector in the provision and delivery of health services, especially in middle- and low-income countries (1−7). The private health sector is wide-ranging and includes traditional and modern systems, for-profit and not-for-profit care providers, and a variety of informal networks (7). Data on the specific components of private health sector are generally lacking, usually leading researchers to lump them together in their analyses (7-9). A systematic review undertaken by Peters *et al*. (7) published in 2004 found that the record of the private sector in delivering preventive health services is ‘mixed’ (i.e. some having positive while others negative or no significant effects at all) while its record in delivering curative services is generally good. The authors highlighted the need for more research towards understanding of the various roles of the private sector in providing health services, particularly in low-resource settings. Data on the role of the private health sector in providing abortion services is particularly deficient. Peters *et al*.'s review reported that, of the 71 out of 700 articles that met the inclusion criteria of the review, only one addressed abortion.

This paper seeks to contribute to the literature on the role of non-governmental organizations (NGOs) in providing abortion services in Nepal, a low-income country. After decades of restriction, the role of NGOs in Nepal significantly expanded in the 1990s following political change and liberalization of the economy (10-12). Since then, the NGO sector has played an increasingly important role in advocacy, creation of awareness, community mobilization, and delivery of both preventive and curative health services. Abortion is a recent policy and programme innovation in the country (13). The combination of this recent development and the growing presence of international as well as local NGOs in the health sector provides an opportune context in which to investigate the role of NGOs in the provision of abortion services.

After decades of very strict anti-abortion laws, abortion was legalized in Nepal in 2002 (13). In 2004, the first legally-authorized abortion services were established within the premises of a well-established women's hospital—Paropakar Maternity and Women's Hospital—also referred to as the Maternity Hospital, in the capital city Kathmandu (14). Subsequently, service-delivery sites have been expanded throughout the country. As of mid-2010, abortion service delivery had been established in 331 facilities, including those operated by the government, NGOs, and the private (for-profit) sector (15). The NGOs included Sunaulo Pariwar Nepal, a local organization of Marie Stopes International (MSI); the Family Planning Association, an affiliate of the International Planned Federation of Family Planning, and a few others. The private-sector facilities included medical colleges, hospitals, and clinics.

Of all the abortion clinics, the two that have experienced the highest number of clients are the Maternity Hospital (MH) clinic located in Thapathali and the MSI clinic located in Chabel. The MSI clinic is a recently-established stand-alone clinic located on a floor of a street building. Although both clinics are located in the capital, the general catchment area of the MSI clinic is one that has seen tremendous growth in terms of population, business, and housing in the last 10 to 15 years. The MH clinic is located in the heart of the city. Both clinics generally follow the national protocol in terms of eligibility of the clients (16).

The two clinics are distinct in several programmatic and operational characteristics. The MH is a public-sector clinic subsidized by the Ministry of Health while the MSI is an NGO clinic (operated through Sunaulo Pariwar Nepal) with many chain clinics throughout the country. Whereas the MH clinic does not use any schemes on a sustained basis to inform potential clients of its services, the MSI clinic employs active marketing and advertising strategies. The two clinics also vary in the fees for their services. The MH clinic charges Rs. 1,000 (US$ 14) while the MSI clinic charges Rs. 1,500 (US$ 20.50). The service fee at MH clinic is exclusive of medication and other charges whereas fee at the MSI clinic also covers check-ups, medicines, and other related expenses. Because of the inclusion and exclusion of charges, the actual difference in cost between the two clinics is, however, expected to be somewhat smaller than it appears. The MSI clinic generally provides services for longer hours than the MH clinic.

Being an NGO operated according to international standards, the MSI clinic enjoys the common social perception that it provides quality services in a more user-friendly setting than does a public-sector clinic. The general perception of higher quality is reflected in actual outcomes as well. A study of 7,386 clients at 27 abortion service-delivery sites, conducted in 2008, found that the clients who received services from MSI clinic had significantly lower rates of complications than among clients at other clinics (17). MSI clinic has a “one-window” service-delivery policy whereby all services are provided within the same clinic, thus making its services fully integrated as well as more convenient. The MH clinic, on the other hand, refers clients to other clinics in the hospital for family planning and any other needs. Being part of a large hospital system, referral adds complexity to the service-delivery process and also increases the waiting period for obtaining additional services.

Very little is known about the similarities and differences between clients of the public and non-public clinics. Do clients of NGO clinics necessarily differ from public clinic clients? If so, in what ways? Does the main contribution of NGO clinics lie in increasing the availability of services or in serving clients whose needs cannot be met by public-sector clinics? To the extent that the clientele of the two types of clinics differ, what implications do these differences have for service delivery? The issues in these questions are examined in this paper. Should the results show that the demographic and socioeconomic factors between the two groups are essentially similar, we will conclude that the provision of services through the NGO sector is contributing mainly to the expansion of the availability of and access to abortion services. Alternatively, if the background characteristics of the two groups are significantly different from each other, this will suggest that the NGO sector is catering to different segments of the population seeking abortion services.

## MATERIALS AND METHODS

The data used in the analysis are from a survey of abortion clients of the MH and MSI clinics. These two facilities were selected because, as mentioned above, these are the leading clinics in their respective sectors in terms of number of clients served.

The eligible respondents for the survey were defined as those who presented themselves at the respective clinics for induced (surgical) abortion services and received the services. Data on the number of clients at the two clinics for six months preceding the study (July-December 2009) were used as the basis for estimating the minimum number of cases required from each clinic. The sample-sizes were determined to be 352 and 703 at the MH and MSI clinic respectively. We stopped recruitment at each clinic at the end of the day when the minimum required numbers were obtained. The study was implemented over a 10-week period from 23 December 2009 to 5 March 2010. Because the total number of cases was proportional to the client load of each study site, the merged sample represented the clients of the two clinics, and no weighting of sample was required in the analysis.

The study protocol was approved by the Nepal Health Research Council (the local Protection of Human Subjects Committee). Seven females conducted the interviews under the supervision of a senior staff member, all of whom were given training on the survey contents and techniques of interviewing with sensitivity to clients seeking abortion counselling and services. Many of the questions in the survey instrument were partially open-ended while some were fully open-ended. The responses to these were coded at a later stage. A statement was read to each potential respondent, and verbal consent was obtained before proceeding with the interview. Only a few women refused to participate in the interview. The interview was conducted either before or after undergoing the abortion, depending on the convenience and availability of time.

We compared the two groups of clients along several variables representing sociodemographic characteristics, fertility preferences, context of unintended pregnancy, reasons for having an abortion, attitude towards future abortion, use of contraceptives and non-use, and choice of a particular clinic. Differences between the clients of the two clinics were tested by chi-square for categorical variables and *t*-test for continuous variables. We also used data from service-records from the two study clinics to assess levels of and trends in utilization of services.

**Figure.**
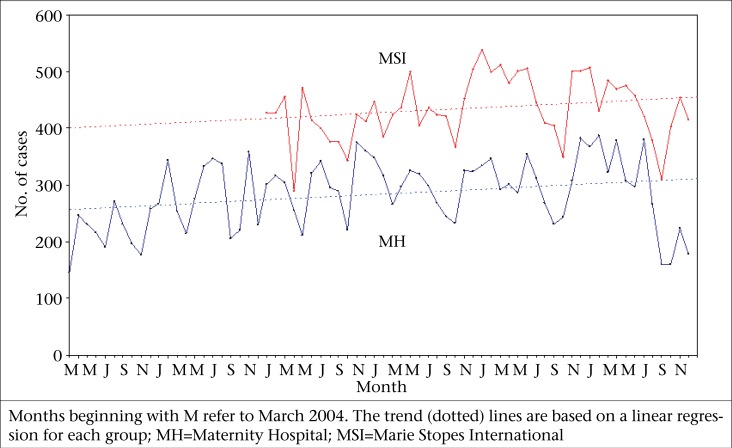
Monthly number of induced abortions performed at the MH clinic (March 2004-December 2009; N=19,798) and MSI clinic (January 2006-December 2009; N=20,879), Nepal

## RESULTS

### Trends in the use of service

The figure shows monthly trends in the number of women who obtained abortion services at the two clinics. Although the MSI clinic had been in operation for a shorter time than the MH clinic (48 vs 70 months), the former had had consistently higher numbers of clients (435 vs 283 on average per month), thus resulting in a higher cumulative number of clients (20,879 vs 19,798) in a considerably shorter period. Despite the fact that the two clinics had different client loads, these were remarkably similar with respect to monthly fluctuations. Generally, the client load was lower in the fall, a season of important festivals, and higher in the winter months. As indicated by the linear regression lines, both clinics have experienced modest overall increases (MSI being relatively higher) in the number of clients over time.

### Clients’ profile

Approximately 31% of the clients in both the groups had previously visited the particular clinic (Table 1). For the majority of the MH (59%) and MSI clients (51%), information about the availability of abortion services came from “friends who had obtained services from the same clinic before.” “Family members or relatives” were the second-most important source of information for the MSI clients (20%). For the overwhelming majority in both the groups, the primary reason for choosing the particular clinic was “availability of good and quality services.” The second-most important reason was “less expensive than other known places” (49% and 64% for the MH and MSI clients respectively). “Convenience/close proximity to home” was cited as another important reason by 40% clients of the MSI clinic. In contrast, proximity was not a very important reason for clients of the MH clinic.

Table 2 shows the demographic and socioeconomic characteristics of the two groups of clients. The average age of the clients was 27.3±5.7 years, with no significant differences between the two groups. Just over one-fourth (26.5%) of the clients had no education, and only about one-fifth had college or higher level of educational attainment. Nearly 50% of the women reported themselves to be housewives not working outside the home. Majority of the clients of MSI clinic lived in households with nuclear family whereas most clients of the public clinic lived in households with joint family.

**Table 1.** Prior visit, source of information, and reason for selecting the clinic among women who had abortions at the MH and MSI clinics, 2010

Variable	MH		MSI		Both
	%	N	%	N	%
Whether ever visited the clinic before^NS^					
Yes	31.6	124	30.5	238	30.9
No	68.4	268	69.5	542	69.1
Total	100.0	392	100.0	780	100.0
Source of information about the availability of abortion services at this clinic					
Through friends who have obtained services before	59.2	232	50.5	394	53.4
Family members/relatives	4.8	19	19.9	155	14.8
None/self	16.3	64	13.2	103	14.2
Read in the newspaper or heard on the radio	6.6	26	6.8	53	6.7
Other health facility/referral	9.2	36	5.1	40	6.5
Other	3.8	15	4.5	35	4.4
Total	100.0	392	100.0	780	100.0
Primary reason for choosing this clinic for services (multiple responses)					
Availability of good and quality services	92.6	363	94.4	736	93.8
Less expensive than other places	48.5	190	63.8	498	58.7
Close proximity to residence/convenience	5.6	22	40.3	314	28.7
Not knowledgeable about other places	9.4	37	5.4	42	6.7
No need to wait for longer duration to get services	14.3	56	0.5	4	5.1
Other	0.5	2	0.3	2	0.3
Total	NA	392	NA	780	NA

In this and subsequent tables, the p value (*p<0.05, **p<01, ***p<0.001) for a given variable refers to test of significance between the MS and MSI samples. χ^2^ and F-tests were performed for categorical and continuous variables respectively; no test was performed for variables with multiple responses; MH=Maternity Hospital; MSI=Marie Stopes International; NA=Not applicable; NS=Not significant at p<0.05

**Table 2.** Demographic and socioeconomic characteristics of women who had abortions at the MH and MSI clinics, 2010

Variable	MH		MSI		Both
	%	N	%	N	%
Age group^NS^					
16-<20 (years)	7.4	29	5.1	40	5.9
20-<25	25.8	101	32.1	250	29.9
25-<30	30.9	121	30.0	234	30.3
30-<35	22.2	87	19.5	152	20.4
35-<40	10.5	41	10.5	82	10.5
40-48	3.3	13	2.8	22	3.0
Total	100.0	392	100.0	780	100.0
Mean^NS^	(27.5±5.8)	392	(27.2±5.6)	780	(27.3± 5.7)
Education^NS^					
Non-illiterate	28.8	113	25.3	197	26.5
Up to primary (grade 1-5)	11.7	46	13.8	108	13.1
Secondary (grade 6-10)	22.2	87	27.2	212	25.5
High school	18.6	73	14.7	115	16.0
College or higher	18.6	73	19.0	148	18.9
Total	100.0	392	100.0	780	100.0
Profession^NS^					
Housewife/not working outside home	48.7	191	48.6	379	48.6
Business	14.0	55	11.8	92	12.5
Government/private-sector service	11.0	43	11.8	92	11.5
Manual work/daily wage	7.4	29	11.3	88	10.0
Student/not working	9.2	36	9.4	73	9.3
Farming	9.7	38	7.2	56	8.0
Total	100.0	392	100.0	780	100.0
Living situation (multiple responses)					
Husband/partner	92.6	363	92.4	721	92.5
Family relative	61.2	240	4.5	35	23.5
In-laws	17.6	69	7.4	58	10.8
Parents/grandparents	3.3	13	2.3	18	2.6
Alone	0.5	2	0.8	6	0.7
Other	0.5	2	2.6	20	1.9
Total	NA	392	NA	780	NA

MH=Maternity Hospital

MSI=Marie Stopes International

NA=Not applicable

NS=Not significant at p<0.05; No test was performed for the variables with multiple responses

Table 3 shows the marital and childbearing status of the clients in the two groups. About 97% reported they were married, with little difference between the two groups. Among the clients of MSI clinic, the proportion that reported unmarried/not engaged was, however, higher than among the clients of MH clinics (3.6% vs 1.0%). The average number of living children was below 2 (1.88±1.08) and did not vary significantly between the two groups. The most common sex composition of the living children was one son and one daughter, followed by one son only. The groups differed in terms of their intention to have another child in the future. Significantly more women in the MH group did not intend to have a (another) child in the future than in the MSI group (63% vs 51%). Further, more women in the MSI group were uncertain with regard to having child in future (12% vs 5%).

**Table 3.** Marital status and childbearing status of women who had abortions at the MH and MSI clinics, 2010

Variable	MH		MSI		Both
	%	N	%	N	%
Marital status**					
Unmarried and not engaged	1.0	4	3.6	28	2.7
Unmarried but engaged	1.0	4	NA	0	0.3
Married	97.4	382	96.3	751	96.7
Divorced/separated	0.3	1	0.1	1	0.2
Widowed	0.3	1	NA	0	0.1
Total	100.0	392	100.0	780	100.0
Whether been pregnant before**					
Yes	87.2	342	81.3	634	83.3
No	12.8	50	18.7	146	16.7
Total	100.0	392	100.0	780	100.0
Among those pregnant before, no. of living children by sex**					
None	NA	0	5.8	37	3.8
One son, one daughter	28.7	98	22.4	142	24.6
One son	19.9	68	21.8	138	21.1
One daughter	14.0	48	12.5	79	13.0
Two sons	13.2	45	11.5	73	12.1
Two daughters	4.7	16	7.1	45	6.3
Two daughters, one son	5.6	19	6.5	41	6.1
Two sons, one daughter	4.1	14	3.6	23	3.8
All others	9.9	34	8.8	56	9.2
Total	100.0	342	100.0	634	100.0
Average no. of living children^NS^	(1.97±1.07)	342	(1.83±1.08)	634	(1.88 ±1.08)
Intention to have a/another child in the future***					
Yes	32.1	126	36.8	287	35.2
No	62.5	245	51.2	399	54.9
Not sure	5.4	21	12.1	94	9.8
Total	100.0	392	100.0	780	100.0

**p<0.01

***p<0.001;

MH=Maternity Hospital

MSI=Marie Stopes International

AN=Not applicable

NS=Not significant at p<0.05

Table 4 shows data on the context and circumstances resulting in the unintended pregnancy and the primary reason for seeking termination. While nearly half of the clients of MSI clinic thought that they would not get pregnant, only about 31% of the clients of MH clinic thought this. More of the clients of MH clinic reported contraception failure than the clients of MSI clinic (33% vs 20%). A substantial proportion in both the groups (28% and 33%) reported “took chance” (i.e. the couples knew they were not doing anything to prevent an unplanned pregnancy but had an unprotected sex anyway) as a reason for the unintended pregnancy. About half of the clients in both the groups mentioned “not wanting any more children” as the primary reason for seeking the termination. The other important reasons cited were “wanting to space having a (another) child” and “work or education.” “Planning to go abroad” was considerably higher among the clients of MSI clinic (8.1% vs 4.1%). The survey asked an open-ended question about the respondent's perception of the potential consequences of having the child instead of terminating the pregnancy. The most frequently-cited reason by both the groups was “inability to afford the next child.” “No time to look after the new child” was the second-most important reason among the clients of MSI clinic. “Social embarrassment/disgrace” was perceived as a consequence by just over one in 10 women in both the groups.

**Table 4.** Circumstances resulting in unintended pregnancy and perceived consequences among women who had abortions at the MH and MSI clinics, 2010

Variable	MH		MSI		Both
	%	N	%	N	%
Situation/circumstances resulting in unintended pregnancy***					
Did not think I would become pregnant	30.9	121	48.8	381	42.8
Took a chance	33.2	130	28.2	220	29.9
Family planning/contraception failed	32.7	128	19.5	152	23.9
Did not plan to have intercourse at all	3.3	13	3.2	25	3.2
Other	NA	0	0.3	2	0.2
Total	100.0	392	100.0	780	100.0
Primary reason for pregnancy termination (multiple responses)					
Have enough children already	53.1	208	49.0	382	50.3
Want to space childbearing	16.1	63	14.9	116	15.3
Work or education	11.2	44	13.2	103	12.5
Cannot afford	11.7	46	7.6	59	9.0
Going abroad	4.1	16	8.1	63	6.7
Not in good health	3.6	14	5.5	43	4.9
Unmarried/recently married	4.6	18	4.5	35	4.5
Husband didn't want the baby	2.3	9	2.1	16	2.1
Other	0.5	2	1.8	14	1.4
Total	NA	392	NA	780	NA
Perceived consequences if had a baby (multiple responses)					
Unable to afford	49.7	195	60.3	470	56.7
No time to look after	9.2	36	17.8	139	14.9
Social embarrassment/disgrace	13.0	51	12.1	94	12.4
Last child too young	13.8	54	10.8	84	11.8
Interference in work/education/travel	8.7	34	12.3	96	11.1
Health effects	4.8	19	7.4	58	6.6
Other	9.7	38	5.5	43	6.9
Total	NA	392	NA	780	NA

***p<0.001;

MH=Maternity Hospital;

MSI=Marie Stopes International;

NA=Not applicable;

No test was performed for variables with multiple responses

Table 5 shows the decision-making process-related responses. Ninety-six percent of the clients of MSI clinic reported to have made the decision jointly. This percentage was somewhat lower for the clients of MH clinic at 83%. About 4% of the clients of MSI clinic and 10% of those of MH clinic reported to have made the decision by themselves first. The clients of MSI clinic came to the decision more quickly than those of MH clinic (5 vs 7 days on average). The clients of MSI clinic had a significantly higher incidence of previous abortion than those of MH clinic (38% vs 21%). Further, the clients of MSI clinic were significantly more likely to state that they would have another abortion again in the future, should the situation arise, than those of MH clinic (48% vs 28%).

Proportionately, more of the clients of MH clinic than those of MSI clinic (53% vs 35%) reported having used contraception in the month of the unintended pregnancy (Table 6). The four methods most-commonly used were: condom, withdrawal, pill, and rhythm. The three-monthly injectables were used by 7% of the clients of MH clinic and 2% of the clients of MSI clinic. Only eight women (four clients in each group) reported using a permanent method (vasectomy or minilap). Women who reported not to have used any method (47% of the clients of MH and 65% of those of MSI clinic) were asked reasons for non-use in an open-ended question. The four most-commonly reported reasons included; actual or perceived health concerns, forgot (either self or partner) to use contraception, disliked (either by self or partner) contraceptive methods, and perception of low risk of pregnancy. More of the clients of MH than MSI clinic cited health concerns and perception of low risk as reasons for non-use. More of the clients of MSI than MH clinic mentioned “forgot to use” and “disliked methods” as reasons for non-use.

**Table 5.** Discussion regarding the decision to have an abortion among women who had abortions at the MH and MSI clinics, 2010

Variable	MH		MSI		Both
	%	N	%	N	%
Primary person who made the termination decision***					
Self	10.2	40	4.0	31	6.1
Person who made pregnant	6.4	25	0.3	2	2.3
Joint decision—self and husband/partner	83.2	326	95.5	745	91.4
Other	0.3	1	0.3	2	0.3
Total	100.0	392	100.0	780	100.0
Time (days) taken to decide to terminate					
Mean***	(6.6±4.5)	392	(4.8±3.7)	780	(5.4±4.1)
Whether any previous abortion***					
Yes	20.9	82	37.9	296	32.3
No	79.1	310	62.1	484	67.7
Total	100.0	392	100.0	780	100.0
Whether would undergo abortion in future***					
Yes	27.8	109	47.7	372	41.0
No	10.2	40	25.0	195	20.1
Uncertain	62.0	243	27.3	213	38.9
Total	100.0	392	100.0	780	100.0

***p<0.001;

MH=Maternity Hospital;

MSI=Marie Stopes International

Nearly all (96%) women in both the groups expressed interest in using contraception in the future (not shown in table). A considerably higher proportion of clients at the MSI clinic (96%) reported receiving full information about various contraceptive methods than at the MH clinic (57%) (Table 6). About 80% of the clients in each group actually left the clinic with some kind of contraceptive. Among those who accepted contraceptives, condom, pill, and injectables were the most common. Among the clients of MSI clinic, nearly one-third accepted condom. Over one-fourth (27.7%-29.8%) of the women in each group left the clinic after receiving injectables. Other methods were accepted by 5%-6% of the two groups.

The survey also asked the study participants about the cost of services and price elasticity (not shown in table). The clients of MSI clinic paid more for the services than those of MH clinic (Rs. 1,495±0.07 vs Rs. 1,285±143, p<0.001). For majority of both the groups of clients (80%-86%), their husband/partner paid for the services. The overwhelming majority (over 90%) in both the groups thought that the cost of services was about right. Further, the clients of MSI clinic seemed to be less sensitive to a hypothetical rise (either by Rs. 200 or Rs. 300) in the cost of services. When asked if they would still go to the same clinic if the fee increased by Rs. 200, 97.9% of the clients of MSI clinic responded affirmatively compared to 79.8% (p<0.001) of the clients of MH clinic. The corresponding percentages at Rs. 300 were 79.3% and 85.3% (p<0.01).

**Table 6.** Use of contraceptives among women who had abortions at the MH and MSI clinics, 2010

Variable	MH		MSI		Both
	%	N	%	N	%
Whether contraceptive used at the time of pregnancy***					
Yes	52.8	207	35.0	273	41.0
No	47.2	185	65.0	507	59.0
Total	100.0	392	100.0	780	100.0
Method used among women who used a contraceptive method*					
Withdrawal	27.1	56	38.5	105	33.5
Condom	29.0	60	29.3	80	29.2
Pill	19.3	40	17.2	47	18.1
Rhythm	15.9	33	11.4	31	13.3
Injectables	6.8	14	2.2	6	4.2
Vasectomy	1.4	3	1.5	4	1.5
Minilap	0.5	1	NA	0	0.2
Total	100.0	207	100.0	273	100.0
Reason for non-use among women who did not use a method (multiple responses)					
Health concerns (actual or perceived)	42.7	79	33.1	168	35.7
Forgot to use—self or husband	13.0	24	24.5	124	21.4
Disliked—self or partner	18.4	34	20.3	103	19.8
Perceived low risk of pregnancy	18.4	34	12.4	63	14.0
Infrequent sex	1.6	3	7.7	39	6.1
Child too small	2.7	5	5.5	28	4.8
Other	6.5	12	5.7	29	5.9
Total	NA	185	NA	507	NA
Whether received full information about various contraceptive methods***					
Yes	57.1	224	95.9	748	82.9
No	42.9	168	4.1	32	17.1
Total	100.0	392	100.0	780	100.0
Contraceptive method dispensed at discharge***					
Condom	20.2	79	32.4	253	28.3
Pill	22.4	88	15.1	118	17.6
Injectables	29.8	117	27.7	216	28.4
Other	5.9	23	4.7	37	5.1
None	21.7	85	20.0	156	20.6
Total	100.0	392	100.0	780	100.0

*p<0.05,

***p<0.001;

MH=Maternity Hospital;

MSI=Marie Stopes International;

NA=Not applicable;

No test was performed for the variables with multiple responses

## DISCUSSION

A client's decision to choose a particular facility for obtaining services depends on several factors and on how a client weighs those factors in her decision-making process. The study design did not include providing the clients with a choice between the two clinics prior to their visit to a particular clinic for abortion services. Rather, the clients self-selected a clinic based on their own prior information and preferences as well as constraints. Some of the variations observed in clients of the two clinics were obviously affected by where the clients lived and by the location of the clinic itself. For example, convenience and close proximity of the clinic to their residence was also an important factor, especially for the clients of NGO (MSI) clinic but less so for those of the public (MH) clinic.

The quality of services offered by the clinic was important for the overwhelming majority of clients of both NGO and public clinics, and they believed that the place they chose offered quality services. For both the groups, cost was also an important consideration for choosing a particular clinic, even more so for clients of the NGO clinic, although they paid higher fees. Clearly, the clients in each group had their own “reference clinics” for cost comparison, and each client chose a particular clinic because of being cheaper than alternative places which she was aware of.

Although the clients using the public and NGO clinics were remarkably similar with respect to age, education, profession, and parity, the clients of the NGO clinic seemed to be in a relatively more transitory stage in their reproductive career than those using the public clinic. More of the clients of NGO clinic intended to have a child sometime in the future and had an abortion in the past, or were willing to consider having one in the future. The proportion of clients who accepted contraceptives upon discharge was about equal for the two groups. Given that the public-sector clinic had proportionately more clients who did not want to have any more child(ren) in the future than the NGO clinic, one would expect a higher percentage of these clients to leave the clinic with a contraceptive method or to be using more effective methods. The fact that not all forms of contraceptives were offered at the MH clinic may, in part, explain why the proportion of clients of MH clinic accepting contraceptives was not higher. It may also be partly because the MH clinic was less thorough than the MSI clinic in providing clients with information on contraceptives. The discrepancy between the data on desire for a child in the future and acceptance of contraceptives upon discharge suggests the importance of devoting attention to understanding and addressing contraceptive needs of clients in the post-abortion counselling process and service delivery.

Developing a more effective referral system and improving linkages between abortion and family planning clinics, particularly for the public-sector clinic, remain critical challenges. In the case of MH clinic, no mechanisms existed at the time of the study for monitoring referred cases (e.g. implants, IUDs, or sterilization). As abortion services have only recently been added to Nepal's healthcare system, these have yet to be fully incorporated and integrated into the national reproductive health programme. Achieving this will require substantial reform of the existing programme. A fuller understanding of the factors impeding improvement of referral processes and coordination of abortion and family planning services is needed, especially in settings, such as the Maternity Hospital where the abortion clinic and family planning clinic remain physically separate.

The data on the use of contraceptives surrounding the time of unintended pregnancy also have implications for counselling and service delivery. Proportionately, more clients of the public clinic reported the use of contraceptives surrounding the time of unintended pregnancy but they also experienced higher rates of failure. The most commonly-used methods among women in both the groups were condom and withdrawal. Of those who had used a contraceptive method, two in five had practised withdrawal, which is known to be considerably less effective than other methods (18). To the extent that clients’ concerns about contraceptives are addressed in the counselling process and the follow-ups assured, the users of less effective methods (e.g. withdrawal or rhythm) may be open to switching to more effective ones, thus resulting in fewer unintended pregnancies and abortions (19). In addition, introducing users of the withdrawal and rhythm methods in particular to Standard Days Method (STM) as an option may also prove useful since some may retain a preference for a ‘natural’ type of birth control. Although STM's real-life effectiveness is only slightly better than the real-life effectiveness of condoms (18), it may be significantly more effective than practising withdrawal or rhythm.

Results of the study revealed that the highest percentage of women accessing abortion at both the clinics were those who had one son and one daughter, followed by one son. This suggests preference for a particular sex composition or a son. Increased access to abortion services may be exacerbating the son preference factor that is common in Nepal as well as in other South Asian countries (20). Although elimination of son preference is recognized often to be a difficult and lengthy process (20,21), the evidence from the data reported here points to the need to monitor the situation, particularly among abortion clients and ensure that effective mechanisms exist to prevent abortion from being used for sex-selective purposes. This is also consistent with Nepal's policy regarding abortion (13).

### Conclusions

The data analyzed in this paper show that both public and NGO clinics were essentially drawing clients from the same large pool of women. The NGO clinic did not necessarily attract clients who were different with respect to basic sociodemographic characteristics. The survey findings, together with the trend data based on service statistics, lead us to conclude that the NGO clinic has contributed principally to expanding the availability of and access to abortion services. The growth in this particular MSI clinic suggests that the strategies adopted in selecting the location, setting prices, and promoting services to potential clients have worked well. In addition to giving a choice of care providers to women seeking abortion services, the NGO clinic has most likely also eased the burden on the public sector.

The clients of the respective clinics differed in their reproductive behaviour, and the differences were not associated with age, parity, or education. Greater attention to fertility goals and clients’ needs for contraceptives is needed in counselling and service delivery. The data also underscore the importance of improving linkages between abortion and family planning clinics, especially in the Maternity Hospital.

Being the leading abortion clinics representing the public and NGO sectors, much of what occurs in the two clinics, as discussed here, affects a large proportion of women accessing abortion services in the country. To this end, the importance of periodically undertaking process evaluation in quality assurance, identifying areas that need strengthening, and, most importantly, addressing gaps in service delivery at the respective clinics cannot be overemphasized.

## ACKNOWLEDGEMENTS

This paper is based on a study funded by Ipas/Nepal through Valley Research Group as part of its support for health services research and programme evaluation. The authors are grateful to Dr. Indira Basnett for her management support throughout all stages of the study and oversight, to Shital Bhandary for his assistance in sample estimation, to Biru Maharjan, Hari Maharjan, Bhima Dulal, Nisha Rana Magar, Sagun Rana, Uma Shrestha, and Yashodha Thapa for interviewing the study participants, to Jaya Poudel for on-site supervision, and to Emily Read for research assistance at the time of preparation of the main report on which this paper is based. The authors, however, are solely responsible for the contents of the paper and interpretation of the data. The findings were presented and their implications discussed at a national symposium in Kathmandu in 2012.
